# Public perception of healthcare system response to COVID-19: Findings from a web based observational study in Villavicencio, Colombia

**DOI:** 10.1371/journal.pgph.0000702

**Published:** 2022-09-28

**Authors:** César García Balaguera, Olga Yesenia García, María Victoria Gutiérrez

**Affiliations:** 1 Public Health, School of Medicine, Cooperative University of Colombia, Villavicencio, Colombia; 2 Research Incubator of the GRIVI Group, School of Medicine, Cooperative University of Colombia, Villavicencio, Colombia; University of Oslo Faculty of Medicine: Universitetet i Oslo Det medisinske fakultet, NORWAY

## Abstract

This study explores the community perceptions of COVID-19 and the healthcare system’s response to it.: A web-based descriptive observational study was conducted on the general population during the third quarter of 2020 through the application of a survey via social media. Of the sample, 55% have minimal connection with prevention programs, while 66.3% received little or no information about COVID-19, and 69.62% were considered at risk of getting sick from COVID-19. Further, 73.14% were afraid to go to healthcare centers fearing the risk of becoming infected by COVID-19. The low-income population is at greater risk (OR 4.32), as well as those who have not been informed by their insurer of the risks of COVID-19 (OR 2.18). There is a need to strengthen the healthcare system and the quality and design of effective self-care educational interventions during the pandemic.

## Introduction

In the context of the COVID-19 pandemic, there is great concern regarding the adequacy of healthcare systems, especially medium and high complexity services, globally. Doubts have arisen about whether there are enough hospital beds, operating rooms, and intensive care units. There is also concern about the availability of rapid and reliable diagnostic tests in addition to the uncertainty about the presence of a trained and safe epidemiological surveillance system; field workers with experience and availability; sufficient trained medical and nursing staff, enjoying job security and stability; effective mechanisms of education and communication with the population; and, above all, a healthcare system that cares for the most vulnerable [1].

The characteristics of the clinical picture of COVID-19 are partially known, and new findings come to light every day. However, it has been ascertained to be not just a pulmonary disease but also a systemic disorder that affects multiple organs and systems, as demonstrated by the study by Richardson’s group, conducted with 5,700 COVID-19 patients in New York, which showed that the disease affects people with comorbidities and of older age more aggressively [2]. Its mechanisms of damage to the host are already known [3,4], and several vaccines have already been approved and are being administered in various countries [5].

The saturation of health services has reached maximum levels during the pandemic and given the high capacity and speed of transmission, it can overwhelm the capacity of health services, which requires contingency plans based on the experience of countries such as China, Korea of the South and the United States [6].

There is high demand for respirators and intensive care for seriously ill patients—whose proportion ranges between 4% and 15% depending on the affected country. This implies the population characteristics of those affected; operability of healthcare systems; availability of trained human resources and high technology for diagnosis and management; and the high social, emotional, and economic cost of the disease [7,8]. In view of this situation, it is necessary to identify community perceptions about the healthcare system response under pandemic conditions due to infectious diseases. The aim in this case is to also understand community perceptions regarding the healthcare system in relation to COVID-19 in Villavicencio Meta, Colombia.

## Materials and methods

### Statement of ethics

As for the ethics committee, in accordance with Colombian law, in line with the ethical criteria described in resolution 8430 of 1993, the study was endorsed by the Ethics Committee of the Universidad Cooperativa de Colombia, through Act 034 of 2020, informed consent was obtained from the participants prior to conducting the online survey.

A descriptive cross-sectional study was designed and conducted through a self-administered online survey, it was disseminated through whatsapp social networks and email, by social leaders from the different communes, who replied to the people who met the inclusion criteria, between July and September 2020 with prior informed consent. It addressed issues of access to services, health promotion programs, prevention of the disease, barriers to access to care, (delay time in health care, free choice of treating physician, knowledge about the benefit plan and level of satisfaction with health care), quality of care, perception of the risk of COVID-19, and health insurance. The inclusion criteria for the study include those who [1] are residents of Villavicencio Meta, Colombia, [2] are of legal age, and [3] have signed the informed consent form. The exclusion criteria include incomplete completion of the survey and not having signed the informed consent.

Sampling was conducted by communes, expecting a prevalence of use of services of 10%, with which a sample of 384 surveys was estimated, with heterogeneity of 50%, a margin of error of 5%, and a confidence level of 95%, 589 surveys were included in the study, which are 153% of the estimated initial sample, and are 2.86% of the total population.

A descriptive cross-sectional observational study of a quantitative nature was conducted with a sample comprising participants from the eight communes of Villavicencio. A pilot test with 2% of the sample was designed to refine the instrument. The surveys were entered in Excel and subsequently analyzed using Stata 12. A descriptive univariate analysis was performed by evaluating the frequencies in the qualitative variables and by estimating the measures of dispersion and central frequency for the quantitative ones. Frequencies were evaluated with 95% chi^2^ test. Multinomial regression was performed to search for possible associations between the risk factors and the outcome variable, which was Polymerase Chain Reaction CRP+ for COVID-19, ORs were obtained.

## Results

In this study, 589 people were surveyed, of whom 540 people who met the inclusion criteria were included, and 49 people were excluded. In Villavicencio, the population is distributed in eight communes in the urban area and eight districts in the rural area. Most respondents are located in communes 7 and 5, and representative samples were taken from all communes, mostly from urban areas.

At the time of the survey, 99.4% (537) of the sample had health insurance, which was not the case in 0.6% (3) of the respondents. In relation to the type of affiliation, 81.3% (438) are covered by a contributory scheme (population with ability to pay), 9.1% (49) are enrolled in the subsidized regime (without ability to pay), 4.8% (26) are affiliated to a special system, and 4.8% of participants (26) do not know which scheme they are affiliated to. It is evident that 50% of the respondents spend more than $200,000 (55.27 USD) in their monthly budget for health insurance purposes. This corresponds to 22% of the minimum wage in Colombia.

### Perceived healthcare quality

It can be observed that 75.7% of those surveyed consider that there is not enough variety of doctors, clinics, and hospitals for utilizing their health insurance. Of the surveyed families, 55.18% stated that their members have minimal or no links to disease prevention and health promotion programs. In addition, 53.8% of those interviewed had never been contacted to join disease prevention and health promotion programs, while 33.14% had been contacted at some time for that purpose. Regarding the information about the benefit plan to which they are affiliated with their insurer, 48.65% are little or not at all informed about it, and 22.77% are very or extremely informed.

As for how informed the participants were about the municipal collective intervention plan, 71.28% responded that they have little or no information about these interventions.

Further, 65% of those surveyed said they are satisfied with their insurer whereas 35% were not. With regard to the medical appointment request mechanism, 52.3% made appointments by phone and 44.07% requested them by going to the service center in person. Of the survey participants, 32.2% stated that they were not free to choose the treating physician or the medical care center. According to 60% of the interviewees, the waiting period for an appointment ranges between 2 and 7 days, whereas 33.13% said they had to wait from 8 to more than 20 days.

Of those surveyed, 35.12% perceived the medical care in their Health promotion company EPS as average, poor, or terrible, and 64.43% believed that they received excellent or good healthcare. According to 52.39% of the participants, the waiting time at emergency care units was from 1 to 6 hours, and 27.4% stated they had to wait for 1 hour or less. The promptness and completeness in the delivery of medications was excellent or good for 50.54% of the interviewees; however, 12.77% referred to it as poor or terrible. The perception of quality of the clinical laboratory service was excellent or good for 72.4% and poor or terrible for 5.36% of the people surveyed. The waiting time for appointments made with a specialty doctor was less than 15 days for 33.88% and more than 15 days for 55.75% of the respondents.

With regard to the level of information that respondents receive from their Health promotion company Benefit plan management company (insurer) EAPB. about procedures, progress of treatment, etc., 58.14% reported that their service is good or excellent, and 41.46% stated that it is average, poor, or terrible. With regard to the perceived barriers to access to health services, Colombia implemented the payment of a monetary fee, known as moderating fee, to attend to medical or dental appointments, except for emergencies, with the aim of moderating the use of healthcare services to avoid collapsing care centers. This payment is made according to each person’s income; however, 24.30% of the participants stated that not being able to pay the moderating fee prevented them from going to medical or dental appointments.

Of those surveyed, 25.06% prefer to use emergency services rather than having to wait many days to go to a scheduled outpatient appointment, 5.38% would rather use emergency services to avoid paying the moderating fee. Further, 67.34% of those referred to a specialist doctor had experienced a waiting time of more than 15 days for their appointment. Of respondents, 65.24% would recommend their HPA to family and friends, whereas 35.80% had considered changing their insurer (see Table 1).

**Table 1:** Perception of barriers to access to health services.

	YES		NO		TOTAL	
VARIABLE	%	n	%	n	%	n
Have you ever not made an appointment or not gone to a medical or dental appointment because you did not want to or could not pay the moderating fee?	24.30	131	75.69	408	100	539
Have you ever gone to the emergency room to avoid a delay in getting an appointment with a general physician?	25.60	138	74.39	401	100	539
Have you ever gone to the emergency room to avoid paying the moderating fee and copayments?	5.38	29	94.61	510	100	539
Have you ever been referred to a specialty doctor?	67.34	363	32.65	176	100	539
Would you recommend your health insurance to your family and friends?	65.24	351	34.75	187	100	538
Have you thought about changing your health insurance?	35.80	193	64.19	346	100	539

### Information about COVID-19

Questions were asked about aspects related to the perception of COVID-19 and the response of the healthcare system. A perception of having received little information about COVID-19 from insurers at the height of the pandemic was noted among the respondents. With regard to the information received by the Benefit plan management company (insurer) EAPB to prevent COVID-19, 66.37% of the respondents reported that the service was average, poor, or terrible, and 30.55% said that it was excellent or average.

Of those surveyed, 69.62% considered themselves at risk of getting sick from COVID-19, and 25.55% considered themselves at risk of being seriously ill or at high risk of dying from COVID-19. Further, 73.14% are afraid to go to healthcare centers for fear of being infected with COVID-19, whereas 49.9% stated that the HPAs would treat them well if they fell ill with COVID-19. However, only 29.49% had been contacted by the EAPB to inform them of the risks of COVID-19 and where to go if they had any symptoms. Of those surveyed, 5.32% claim to have tested positive for COVID-19 by a PCR test (see Table 2).

**Table 2:** Information received about COVID-19.

	YES		NO		TOTAL	
VARIABLE	%	n	%	n	%	n
Do you consider yourself at risk of getting sick from COVID-19?	69.62	376	30.37	164	100	540
If you get sick from COVID-19, do you consider yourself at risk of being seriously ill or at risk of dying?	25.55	138	74.44	402	100	540
Have you or someone in your family been afraid to attend your EAPB for fear of being infected with COVID-19?	73.14	395	26.85	145	100	540
Do you consider that the health service you are enrolled in would effectively treat you well, if you were to get sick with COVID-19?	49.90	268	50.09	269	100	537
Has your EAPB contacted you to inform you of the risks of COVID-19 and where to go if you have symptoms?	29.49	159	70.50	380	100	539
Have you been tested positive for COVID-19 through a laboratory analysis?	5.32	28	94.67	498	100	526

As for their perception, 61.11% of respondents who had been infected with COVID-19 reported that the service received from their EAPB was average, poor, or terrible, and 38.88% stated that it was excellent or good.

### Bivariate analysis

#### Risk factors

A bivariate analysis was conducted with a 2x2 table, calculating odds ratio (OR) of prevalence, seeking the most possible associations of causality, to be evaluated in subsequent analytical studies. Having had a PCR+ report for COVID-19 was considered an outcome variable, whereas the exposure variable was being a population with no ability to pay, insured by the state subsidy system, which was crosschecked against the variables listed in Table 3.

**Table 3:** Bivariate analysis.

Variable	RISK FACTOR	ODDS RATIO (OR)	CONFIDENCE INTERVAL	P
How much of your monthly budget do you spend on your EAPB health insurance?	**No ability to pay**	4.3269	**OR (1.4235–13.1520)**	0.0052
Would you change your health service provider institution (HPI) or your treating physician?	**Yes**	0.5671	OR(0.3297–0.9756)	0.0386
What percentage of your family is connected by the EAPB to a promotion and prevention program (healthy youth, oral health and hygiene, diabetes, breast cancer, hypertension, obesity, prenatal care, etc.)?	**Less than 20%**	0.5624	OR (0.3220–0.9823)	0.0412
Level of information on the healthcare services of the Collective Intervention Plan (PIC, for its Spanish acronym) to be provided by your municipality (the Municipal Mayor’s Office).	**Little**	1.8827	**OR (1.0192–3.4779)**	0.0407
When your appointment is scheduled, the time between your request and the day of the consultation is:	**Greater than 3 days**	0.5665	OR (0.3441–0.9328)	0.0243
Have you been to the emergency room to avoid a delay in getting an appointment with a general practitioner?	**Yes**	2.7656	**OR (1.6193–4.7233)**	0.0001
Has your EAPB contacted you to inform you of the risks of COVID-19 and where to go if you have symptoms?	**No**	4.080	OR (0.2135–07795)	0.0053
The information you received from your EAPB to prevent becoming infected by COVID-19 is:	**Poor**	2.1879	**OR(1.1632–4.1152)**	0.0132

Benefit plan management company (insurer) EAPB.

The main risk factors found in the study are being a population without the ability to pay (OR 4.32, CI 1.42–13.15), having little information about the municipal plan of collective interventions focused on health promotion and disease prevention (OR 1.88, CI 1.01–3.47), having gone to the emergency room because of the delay in scheduling an outpatient appointment (OR 2.76, CI 1.71–4.72), and having received little information from their insurer about the prevention of COVID-19 (OR 2.18, CI 1.16–4.11) (see Table 3).

## Discussion

This study’s results reveal that the respondents’ perception of the healthcare system was of it not being well-equipped to face a pandemic like COVID-19. Insurers do not comply with fundamental factors included in the Colombian legislation, as in the case of not respecting the patients’ freedom of choosing the healthcare service provider and the treating professional, a sensitive aspect for 75.7% of the interviewees. Studies conducted in China, Italy, and Ethiopia had already found similar results that impact public trust in healthcare and make people seek care at very serious stages of the disease [9–15].

Another crucial element has to do with connecting the population to health promotion and disease prevention programs; not more than 55% of those surveyed were connected to such programs in contrast to studies conducted in England, Egypt, Germany, Italy, and Ethiopia, where this connection is a great strength in educational and preventive measures for COVID-19, other pathologies, and endemic risks in populations [13,14,16–18].

The majority of the surveyed population (48.65%) is poorly or not at all informed of the benefits of being enrolled in the healthcare system, and they are also unaware of the municipality’s plan of collective interventions aimed at preventing disease and promoting health, thereby losing opportunities to achieve a better level of healthcare for populations.

It is evident that 50% of the respondents spend more than $200,000 (55.27 USD) in their monthly budget for health insurance purposes. This corresponds to 22% of the minimum wage in Colombia, these costs are high for a middle or lower class family in Colombia, generating an economic barrier for health care.

Of the interviewees, 60% consider the timeliness of care to be good in outpatient appointments. However, 55.75% found it to be poor for in the case of specialist appointments, with a waiting period of over 15 days; this leads to greater complications and is a barrier to timely preventive interventions, as noted in the Zikargae study conducted in Ethiopia [15].

With regard to COVID-19, the information received by health promotion and disease prevention insurers is very low for 67.37% of the respondents. 69.62% consider themselves at risk of becoming ill, and 25.5% of dying or being very seriously ill, similar to what was found in Saudi Arabia, Sudan, Pakistan, and Finland, with a very similar global perception of risk [19–24]. The low level of confidence in the healthcare system in the event of being infected by COVID-19 stands out, with only 49% stating that they would be well cared for in the event of getting sick.

In conclusion, the perceived fragility of the healthcare system, the insufficient training, information, and communication actions addressed to the population and their expectations regarding healthcare in the COVID-19 pandemic are evident. Therefore, it is necessary to strengthen health promotion and disease prevention actions, improve the timeliness of healthcare, and eliminate care barriers to access services, which have been proven to be effective strategies in various countries [15,25–27].
